# Respect for Patients’ Rights in Hospital Practice: Associations with Physicians’ Legal Knowledge, Defensive Medicine, and Patient-Centered Care

**DOI:** 10.3390/healthcare14193332

**Published:** 2026-10-06

**Authors:** Alina Doina Tănase, Sorin Dan Chiriac, Ștefania Dinu, Adriana Pădure, Codruța-Eliza Ille, Bogdan Hoinoiu, Cristina Ioana Talpoș Niculescu

**Affiliations:** 1 Department of Professional Legislation in Dental Medicine, Faculty of Dental Medicine, “Victor Babeș” University of Medicine and Pharmacy, Eftimie Murgu Square No. 2, 300041 Timisoara, Romania; tanase.alina@umft.ro; 2 Methodological and Research Center Medical Law, Medical Malpractice and Research Ethics, Faculty of Dental Medicine, “Victor Babeș” University of Medicine and Pharmacy, Eftimie Murgu Square No. 2, 300041 Timisoara, Romania; adriana.padure@umft.ro (A.P.); ioana.talpos-niculescu@umft.ro (C.I.T.N.); 3 University Clinic of Surgery III, Faculty of Medicine, “Victor Babeș” University of Medicine and Pharmacy, Eftimie Murgu Square No. 2, 300041 Timisoara, Romania; chiriac.sorin@umft.ro; 4 Pediatric Dentistry Research Center (Pedo-Research), Department of Pediatric Dentistry, Faculty of Dental Medicine, “Victor Babeș” University of Medicine and Pharmacy, Eftimie Murgu Square No. 2, 300041 Timisoara, Romania; 5 Department of Oro-dental Diagnosis and Ergonomics, Faculty of Dental Medicine, “Victor Babeș” University of Medicine and Pharmacy, Eftimie Murgu Square No. 2, 300041 Timisoara, Romania; 6 Faculty of Dental Medicine, “Victor Babeș” University of Medicine and Pharmacy, Revoluției Avenue 1989, No. 9, 300580 Timisoara, Romania; ille.codruta@umft.ro; 7 Research Center of Digital and Advanced Technique for Endodontic, Restorative and Prosthetic Treatment (TADERP), “Victor Babeș” University of Medicine and Pharmacy, Revoluției Avenue 1989, No. 9, 300580 Timisoara, Romania; 8 Department of Oral Rehabilitation and Dental Emergencies, Faculty of Dentistry, “Victor Babeș” University of Medicine and Pharmacy, 300041 Timisoara, Romania; hoinoiu@umft.ro; 9 Interdisciplinary Research Center for Dental Medical Research, Lasers and Innovative Technologies, 300041 Timisoara, Romania

**Keywords:** patient rights, physicians, defensive medicine, patient-centered care, health knowledge, attitudes, practice

## Abstract

*Background/Objectives:* This study examined associations of physicians’ legal knowledge, defensive medicine, and patient-centered orientation with self-reported respect for patients’ rights. *Methods:* A single-center cross-sectional study (February–June 2026) included 96 physicians. The primary outcome was the Patients’ Rights Respect Score (PRRS, 0–100). The confirmatory analysis compared PRRS between legal-knowledge groups (adequate ≥70/100). Multivariable regression was secondary; mediation and prediction were exploratory. *Results:* Adequate legal knowledge (34/96, 35.4%) was associated with higher PRRS (76.8 ± 7.1 vs. 71.0 ± 8.4; difference 5.8, 95% CI 2.6–9.0, *p* < 0.001, d = 0.73) and lower Defensive Medicine Scale scores (44.5 ± 6.8 vs. 48.5 ± 9.3, *p* = 0.016). Patient–Practitioner Orientation Scale scores were independently associated with PRRS (B = 6.64 per point, *p* < 0.001; model R^2^ = 0.513); the adjusted legal-knowledge coefficient was not statistically significant (B = 1.09 per 10 points, *p* = 0.094). In exploratory parallel mediation models, 41.8% of the knowledge–PRRS association was statistically accounted for by indirect associations, mainly through patient-centered orientation; concurrent measurement precludes causal ordering. An exploratory model for PRRS < 70 had an optimism-corrected AUC of 0.789 (internal validation only). *Conclusions:* Legal knowledge was associated with greater self-reported respect in unadjusted comparisons. Indirect associations were hypothesis-generating, and predictive performance does not establish clinical utility. Confirmation requires multicenter, longitudinal studies with objective measures of practice.

## 1. Introduction

Respect for patients’ rights—informed consent, confidentiality, access to information, and the protection of dignity and privacy—has evolved from an ethical aspiration into a codified legal obligation across most health systems. In Europe, dedicated patients’ rights legislation and institutional mechanisms such as patient ombudsmen have been implemented in numerous countries, although the effectiveness of these instruments in changing bedside practice remains uneven [1]. Hospital care concentrates the situations in which these rights are most vulnerable: invasive procedures requiring valid consent, multidisciplinary information flows that strain confidentiality, and acute illness that threatens dignity. Evidence from Central and Eastern Europe indicates that even where health professionals report high awareness of patients’ rights law, translation into consistent daily practice lags considerably behind [2].

The empirical literature documents persistent gaps on both sides of the clinical encounter. Surveys of hospitalized patients have found strikingly low awareness of formal rights regulations, with fewer than one in ten inpatients in some settings knowing that a dedicated patients’ rights framework existed [3]. Studies examining how rights are exercised during routine hospitalization similarly describe incomplete information provision, variable consent quality, and inconsistent protection of privacy, even in systems with long-standing legal guarantees [4]. Because patients frequently cannot verify whether their rights are being honored, the professional attitudes and competencies of physicians become the decisive safeguard, making the determinants of physicians’ rights-respecting behavior an important and measurable quality target.

One plausible determinant is legal knowledge. Physicians’ command of the law that governs their practice is frequently limited: senior specialists answered barely half of basic medico-legal questions correctly in an Australian multistate survey [5], and comparable deficits in knowledge of healthcare law have been described among hospital doctors and nurses in other settings [6]. Reviews of legal education in medical curricula conclude that teaching is heterogeneous, rarely assessed against behavioral outcomes, and insufficient to guarantee legal literacy at the point of care [7]. Whether measured legal knowledge actually tracks with rights-respecting clinical behavior—rather than merely with examination performance—remains an open empirical question.

A second, potentially countervailing influence is defensive medicine. Landmark survey data from high-liability environments show that the overwhelming majority of specialist physicians report assurance behaviors such as unnecessary testing or avoidance behaviors such as declining high-risk patients [8], and more than three-quarters of hospital doctors in the United Kingdom acknowledged practicing defensively [9]. Beyond its economic cost, defensive medicine reorients clinical decisions toward the physician’s legal self-protection and away from the patient’s interests, eroding trust and potentially subordinating autonomy, information sharing, and shared decision-making to documentation and risk management [10].

Patient-centered care offers a third, positive pathway. Observational and trial evidence links patient-centered communication with better recovery, improved emotional health, and fewer diagnostic tests and referrals [11]. The Patient–Practitioner Orientation Scale (PPOS) operationalizes this construct along sharing and caring dimensions and has become a standard measure of whether clinicians favor collaborative, patient-oriented care over disease- and doctor-centered styles [12]. Conceptually, patient-centered orientation may be the attitudinal channel through which abstract legal knowledge becomes concrete rights-respecting behavior, yet these three constructs—legal knowledge, defensive practice, and patient-centeredness—have rarely been measured together.

A conceptual model linking these constructs can be drawn from the knowledge–attitude–practice framework and the theory of planned behavior, in which behavior is shaped by attitudes toward the behavior, perceived norms, and perceived behavioral control [13]. Within this model, legal knowledge is expected to relate to rights-respecting practice through two routes. First, knowing what the law requires (for example, that consent must be informed and voluntary, or that disclosure to relatives requires the patient’s agreement) reframes patients’ rights from discretionary courtesies into professional obligations, which is expected to strengthen a patient-centered attitude that values information sharing and participation; this attitude, in turn, is expected to be enacted as rights-respecting behavior. Second, accurate knowledge of legal requirements should reduce the uncertainty and perceived litigation threat that motivate defensive practice [14], which competes with patients’ interests for the physician’s attention. Legal knowledge may also act directly, by supplying the procedural know-how needed to implement a right, independently of attitude. Because attitudes may equally motivate physicians to acquire legal knowledge, and because all constructs were measured concurrently, the present study can test whether the data are compatible with this model but cannot establish its causal direction.

Existing studies have examined these constructs largely in isolation: legal knowledge is typically assessed against examination-type criteria [5,6,7,15,16], defensive medicine is quantified as a prevalence [8,9,14], and patient-centered orientation is related to satisfaction or communication outcomes [11,12], whereas the outcome that ultimately matters to patients—whether their rights are respected in practice—is seldom measured alongside them; recent Romanian data indicate that respect for patients’ rights in routine and crisis practice remains incomplete [17]. To our knowledge, no study has jointly quantified physicians’ legal knowledge, defensive medicine, and patient-centered orientation as correlates of an integrated measure of respect for patients’ rights, nor examined whether knowledge relates to practice indirectly through attitudes or differently across professional subgroups. Clarifying these associations may inform hypotheses about interventions targeting legal instruction, attitudes, or the defensive climate. The objective was to estimate the association between physicians’ legal knowledge and the degree of respect for patients’ rights in hospital practice, and, in exploratory analyses, to characterize the roles of defensive medicine and patient-centered care as correlates, statistical mediators, and moderators of that association.

## 2. Materials and Methods

### 2.1. Study Design and Setting

We conducted a cross-sectional, questionnaire-based study, with data collection for the period from 3 February to 26 June 2026. The study was designed, analyzed, and reported in accordance with the Strengthening the Reporting of Observational Studies in Epidemiology (STROBE) recommendations for cross-sectional studies [18]. The setting comprised medical, surgical, and emergency/intensive care departments of an academic hospital, reflecting the case mix in which patients’ rights issues most commonly arise. The unit of observation was the individual physician, and the PRRS and DMS referred to self-reported practice during the preceding six months. All instruments were administered in Romanian.

### 2.2. Participants, Eligibility Criteria, and Group Allocation

Eligibility criteria were defined a priori. Inclusion criteria were: (i) physicians (residents, specialists, or senior physicians) with clinical activity involving direct inpatient contact; (ii) at least one full year of clinical practice in the hospital; and (iii) provision of informed consent to participate. Exclusion criteria were: (i) exclusively administrative, laboratory, or imaging activity without direct patient care; (ii) absence from clinical duty for more than three of the preceding six months; and (iii) questionnaires with more than 10% missing items. Eligible physicians (*n* = 121) were identified from departmental staff lists, and each received a single-use, non-identifying access link to the anonymous electronic questionnaire; the response rate was calculated as the number of submitted questionnaires (104; 86.0%) divided by the number of links distributed, without any linkage between links and individual identities. Eight questionnaires were excluded for exceeding the missing-data threshold, yielding an analytic sample of 96 participants. Because the excluded questionnaires contained the completed demographic section, the sex distribution of excluded and retained respondents could be compared (Section 3.1).

Participants were allocated to two comparison groups defined a priori by performance on the legal knowledge test: adequate legal knowledge (score ≥ 70 of 100, *n* = 34) and inadequate legal knowledge (score < 70, *n* = 62), a threshold aligned with the pass standard commonly used for professional licensing assessments. Pre-specified subgroup analyses considered sex, specialty profile (surgical vs. non-surgical), clinical experience (<10 vs. ≥10 years), recent legal training (structured medico-legal course within five years), and prior malpractice complaint exposure. The sample size target was set before recruitment for the primary comparison of PRRS between the two knowledge groups: assuming, from prior Romanian data [15], that approximately one-third of physicians would reach the adequacy threshold (1:2 allocation) and that a standardized mean difference of d = 0.60 would be the smallest difference in practical importance, the recruitment target was 90 participants (30/60), and 121 physicians were invited to allow for non-response and exclusions. A check of the planning assumptions using a two-sided noncentral t distribution gives approximately 76% power for 30/60 participants at d = 0.60 and α = 0.05. The secondary multivariable model included eight predictors in 96 participants; its estimates require cautious interpretation. The interaction, mediation, spline, and predictive analyses were not powered a priori: interaction tests in samples of this size have low power, and the logistic prediction model, with 33 events and eight predictors, had approximately four events per variable, well below the 10–20 recommended for stable estimation [19,20]. These analyses were therefore prespecified as exploratory and hypothesis-generating (Section 2.4).

### 2.3. Variables, Outcomes, and Data Collection

The primary outcome was the Patients’ Rights Respect Score (PRRS), a 24-item self-report instrument developed for this study, covering four domains derived from the Romanian Patients’ Rights Law (Law No. 46/2003) and the European Charter of Patients’ Rights: informed consent, confidentiality, right to information, and dignity and privacy (six items per domain). Each item asks how often, during the preceding six months, the physician performed a specified rights-respecting action (e.g., explaining the risks, benefits, and alternatives of a proposed procedure before obtaining consent), rated on a five-point frequency scale (never, rarely, sometimes, usually, always) and rescored as 0, 25, 50, 75, and 100. Each domain was scored 0–100 as the mean of its items, and the total PRRS was the mean of the four domain scores, with higher values indicating self-reported practice more respectful of patients’ rights. The PRRS had not been used in a previous study; its development and preliminary psychometric evaluation are summarized below. Legal knowledge was assessed with a 25-item multiple-choice test addressing statutory provisions on consent, confidentiality, information disclosure, treatment refusal, and the rights of minors, scored as the percentage of correct answers (0–100), constructed for this study from the same legislation by two medical-law specialists and reviewed by an independent expert panel; the items test knowledge of statutory rules (e.g., the age of consent for minors, the conditions under which confidentiality may lawfully be breached) rather than self-reported behavior. Defensive medicine was measured with a 15-item Defensive Medicine Scale (DMS; total 15–75; items scored 1–5 by frequency of practice) adapted from the behavior inventories used in previous physician surveys of defensive medicine [8,9,21], with defensive practice defined in the narrower sense of deviation from sound practice motivated by fear of litigation or complaint [14], comprising assurance (9 items, 9–45) and avoidance (6 items, 6–30) behavior subscales. All 15 DMS items were administered in Romanian using the study-specific adaptation of the cited behavior inventories. Patient-centered orientation was measured with the 18-item Patient–Practitioner Orientation Scale (PPOS; 1–6), including Sharing and Caring subscales [12], administered in a Romanian version produced by forward and backward translation with reconciliation by a bilingual committee, as no published Romanian validation was available; higher scores indicate a more patient-centered orientation.

The PRRS underwent expert content review and pilot testing; the scale-level content validity index (S-CVI/Ave) was 0.91 [22]. In the analytic sample, internal consistency was high for the total score (Cronbach’s α = 0.89) and acceptable for the four domains (α = 0.79–0.85). The preliminary factor analysis suggested four correlated domains, but the sample was small (four participants per item) and external criterion validity remains unestablished. Reliability of the other instruments in the present sample was as follows: legal knowledge test, Kuder–Richardson 20 = 0.81; DMS, α = 0.86 (assurance α = 0.84, avoidance α = 0.77); PPOS, α = 0.80 (Sharing α = 0.74, Caring α = 0.72). The full PRRS items and the legal knowledge test items, with the answer key and the statutory provision addressed by each item, are available from the corresponding author upon reasonable request in English translation of the Romanian versions administered.

Secondary outcomes comprised the four PRRS domains, DMS total and subscale scores, PPOS scores, and six binary rights-related practice indicators (e.g., always obtaining written consent before invasive procedures, verifying patient understanding through teach-back, and awareness of the hospital patients’ rights procedure). Covariates were age, sex, specialty profile, professional grade, years of experience, recent legal training, and prior malpractice complaints. Demographic data also recorded whether the physician had completed a formal medical law or ethics course during undergraduate or residency training and the number of hours of medico-legal training in the preceding five years. Data were collected through an anonymous electronic questionnaire; anonymity was a condition of ethical approval and of participation, which precluded linkage of responses to chart audits, patient-reported experiences, or observational assessment of the same physicians (see Section 4.2). The a priori missing-data rule specified person-mean imputation for scales with <10% missing items and exclusion otherwise; 8 questionnaires were excluded for exceeding the missing-data threshold, and all analyses used the complete analytic set of 96 records.

### 2.4. Statistical Analysis

Continuous variables are summarized as mean ± standard deviation (SD) or median with interquartile range (IQR), according to distribution, and categorical variables as counts and percentages. Normality was assessed with the Shapiro–Wilk test. Between-group comparisons used Welch’s unequal-variance *t*-test for approximately normal variables and the Mann–Whitney U test otherwise; categorical outcomes were compared with the chi-square test (Fisher’s exact test when expected counts were <5), with effect sizes expressed as Cohen’s d and odds ratios (OR) with Woolf 95% confidence intervals (CI). Pairwise associations used Pearson correlation coefficients, with Spearman coefficients computed as sensitivity checks. All tests were two-tailed with α = 0.05, and exact *p*-values are reported (*p* < 0.001 when below that threshold), without adjustment for multiplicity; subgroup and secondary findings are interpreted as exploratory.

Analyses were organized in a prespecified hierarchy. The single confirmatory hypothesis test was the comparison of total PRRS between the adequate- and inadequate-knowledge groups. Prespecified secondary analyses comprised the four PRRS domains, the DMS and PPOS scores, the six practice indicators, the correlation matrix, and the multivariable regression of PRRS on legal knowledge and covariates. Prespecified exploratory analyses comprised the subgroup comparisons, the moderation, mediation, and spline analyses, and the predictive modeling with internal validation. Post hoc analyses, undertaken during peer review, comprised the comparison of excluded and retained respondents, the DeLong comparisons of AUCs between nested models, the sensitivity analysis of the suboptimal-PRRS threshold, the sensitivity model including professional grade, and the psychometric analyses of the PRRS. No adjustment for multiplicity was applied; approximately 40 secondary and exploratory tests were performed, so about two nominally significant results at α = 0.05 would be expected by chance alone if all tested null hypotheses were true, and individual secondary findings should be interpreted accordingly. The primary result remained significant under a Bonferroni correction for the five PRRS outcomes (α = 0.01).

Multivariable ordinary least squares regression modeled PRRS on legal knowledge (per 10 points), PPOS, DMS (per 10 points), legal training, malpractice exposure, specialty, experience (per 10 years), and sex. Covariates were selected a priori on conceptual grounds as plausible common causes of legal knowledge and rights-respecting practice or as established correlates of the outcome: clinical experience (accumulation of both legal knowledge and practice habits), sex, surgical specialty (higher frequency of consent-intensive procedures and higher malpractice exposure [23]), recent legal training (a direct determinant of knowledge), and prior malpractice complaints (a driver of defensive practice [21,24]); PPOS and DMS were entered as the hypothesized intermediate variables. Age was excluded because of collinearity with experience (r = 0.968), and professional grade was not entered because it is largely determined by experience in the Romanian career structure (Spearman ρ = 0.81 in this sample); adding grade in a post hoc model changed no coefficient by more than 0.3 points. Model assumptions were verified through variance inflation factors (all ≤1.94), the Breusch–Pagan test, and Shapiro–Wilk testing of residuals, and model performance is reported as R^2^, adjusted R^2^, F statistic, and root mean square error (RMSE). Moderation was examined hierarchically by adding legal knowledge × legal training and legal knowledge × specialty interaction terms to main-effects models, with simple slopes derived from the interaction models. Parallel mediation of the knowledge–PRRS association through PPOS and DMS was tested with percentile bootstrap confidence intervals (5000 resamples), following established resampling recommendations for indirect effects [25]. Mediation and moderation analyses were prespecified as exploratory: because the exposure, mediators, and outcome were measured concurrently, the terms “direct”, “indirect”, and “proportion mediated” are used in their statistical sense and describe the decomposition of a cross-sectional association, not causal effects. The shape of the knowledge–PRRS association was modeled with a restricted cubic spline (four knots at the 5th, 35th, 65th, and 95th percentiles), testing non-linearity by an F-test against the linear model. Finally, exploratory logistic models predicting suboptimal respect for patients’ rights (PRRS < 70) were evaluated with the area under the receiver-operating-characteristic curve (AUC; DeLong 95% CI), the Youden-optimal cut-off, the Hosmer–Lemeshow test, the Brier score, and decision-curve analysis, with internal validation by bootstrap optimism correction (500 resamples) of the AUC and calibration slope. The PRRS threshold of 70 was specified before analysis as an operational cut-off below the “usually” response anchor (75 points), not as a validated clinical or legal boundary. Because PRRS is an average of frequency scores, a score of 70 does not indicate that rights were respected on 70% of items. The numerical agreement with the legal-knowledge threshold does not establish equivalent meaning for the two instruments. Its proximity to the observed lower tertile (33rd percentile, 70.4) is a descriptive finding, not an a priori justification. Because the threshold is operational rather than externally validated, the predictive analyses were repeated with alternative cut-offs (PRRS < 65 and PRRS < 75) as a sensitivity analysis. Three nested models were compared: legal knowledge alone; legal knowledge plus PPOS and DMS; and the full model containing the eight predictors of the multivariable regression (legal knowledge, PPOS, DMS, recent legal training, prior malpractice complaint, surgical specialty, experience, and sex). Differences in AUC between nested models were tested with DeLong’s method for correlated ROC curves. With 33 events, the full model had approximately four events per predictor; its performance is reported as internal validation only and is not proposed for clinical application. Analyses were performed in Python 3.11 (NumPy 2.4, SciPy 1.17, statsmodels 0.14, scikit-learn 1.8).

## 3. Results

### 3.1. Baseline Characteristics of the Study Cohort

Baseline characteristics are summarized in Table 1. Of 96 physicians, 34 (35.4%) had adequate legal knowledge and 62 (64.6%) did not. The adequate-knowledge group had greater median clinical experience (14.5 vs. 10.0 years, *p* = 0.009) and more frequent recent legal training (67.6% vs. 32.3%, *p* < 0.001); sex distribution and prior malpractice complaints did not differ significantly. The adequate-knowledge group was also older (median 43.0 vs. 38.5 years, *p* = 0.019), consistent with its greater experience; because age and experience were almost collinear (r = 0.968), only experience was carried into the multivariable model. A formal medical law or ethics course during undergraduate or residency training was reported by 71 physicians (74.0%), more often in the adequate-knowledge group (85.3% vs. 67.7%, *p* = 0.061), and the median number of medico-legal training hours in the preceding five years was 8 (IQR 0–16) versus 0 (IQR 0–8) (*p* = 0.003). Female physicians accounted for 5/8 (62.5%) excluded respondents and 54/96 (56.2%) retained respondents (two-sided Fisher’s exact test, *p* = 1.000). This small comparison does not establish equivalence between groups or exclude selection bias.

### 3.2. Primary Outcome: Respect for Patients’ Rights

Primary outcome results are presented in Table 2. Mean PRRS was 76.8 ± 7.1 in the adequate-knowledge group and 71.0 ± 8.4 in the inadequate-knowledge group (mean difference 5.8, 95% CI 2.6–9.0; *p* < 0.001). The largest domain difference was observed for dignity and privacy (7.3 points, *p* < 0.001), followed by informed consent (6.1 points, *p* = 0.002) and right to information (5.8 points, *p* = 0.004).

### 3.3. Secondary Outcomes: Defensive Medicine and Patient-Centered Orientation

Secondary outcomes are summarized in Table 3. The adequate-knowledge group had a lower DMS total score (44.5 ± 6.8 vs. 48.5 ± 9.3, *p* = 0.016), with a nominally significant difference in assurance behaviors (*p* = 0.016) but not avoidance behaviors (*p* = 0.082). PPOS total and subscale differences were not statistically significant. The absence of a significant PPOS difference between the dichotomized knowledge groups is not inconsistent with the mediation results in Section 3.9, which use the continuous knowledge score: PPOS correlated with legal knowledge across its full range (r = 0.319, *p* = 0.002; Section 3.6), and dichotomizing knowledge at 70 discards this within-group variation and reduces power, so the two analyses address the same, directionally consistent association with different precision.

### 3.4. Rights-Related Practice Indicators

Rights-related practice indicators are presented in Table 4. Written informed consent was reported by 82.4% versus 69.4%, teach-back by 50.0% versus 37.1%, and disclosure to family without patient consent by 32.4% versus 46.8% in the adequate- and inadequate-knowledge groups, respectively. None of the six individual comparisons reached statistical significance; most point estimates favored the adequate-knowledge group, while documented informed refusal was essentially identical (67.6% vs. 67.7%), and the wide confidence intervals (e.g., OR 0.73–5.80 for written consent) indicate that these binary indicators were imprecisely estimated rather than demonstrably unrelated to knowledge.

### 3.5. Subgroup Analyses

Pre-specified, exploratory subgroup analyses are reported in Table 5, which presents unadjusted within-stratum comparisons; the corresponding covariate-adjusted estimates are shown in Section 3.8. PRRS differences favored adequate legal knowledge in every examined stratum, ranging from 2.0 to 7.4 points, and no interaction test was statistically significant. The largest differences were observed among surgical physicians (7.4 points, *p* = 0.003) and physicians without recent legal training (6.7 points, *p* = 0.041). The adjusted estimates in Section 3.8 were attenuated relative to the unadjusted values (e.g., female sex 4.8 points, *p* = 0.059; non-surgical specialty 3.4 points, *p* = 0.167), whereas the interaction *p*-values are identical in Table 5 and Section 3.8 because both derive from the same interaction term in the adjusted model. The strata of physicians with <10 years of experience (8 vs. 30) and with prior malpractice exposure (6 vs. 14) contained too few adequate-knowledge physicians to support interpretation and are shown for completeness only.

### 3.6. Correlation Analysis

Correlations among the principal continuous measures are shown in Table 6. Legal knowledge correlated positively with PRRS (r = 0.479, *p* < 0.001) and PPOS (r = 0.319, *p* = 0.002) and negatively with DMS (r = −0.320, *p* = 0.001). PRRS correlated positively with PPOS (r = 0.499, *p* < 0.001) and negatively with DMS (r = −0.427, *p* < 0.001).

### 3.7. Multivariable Analysis and Knowledge–PRRS Association

The multivariable regression results are presented in Table 7, and the adjusted knowledge–PRRS association is shown in Figure 1. The model had R^2^ = 0.513 and adjusted R^2^ = 0.468 (F(8, 87) = 11.46, *p* < 0.001; RMSE = 6.1 points). Residuals were homoscedastic (Breusch–Pagan χ^2^(8) = 9.84, *p* = 0.276) and approximately normal (Shapiro–Wilk W = 0.985, *p* = 0.34), and all variance inflation factors were ≤1.94. PPOS was associated with PRRS (B = 6.64 per point, *p* < 0.001), as were recent legal training (B = 3.89, *p* = 0.005) and experience (B = 1.76 per 10 years, *p* = 0.032); the adjusted legal-knowledge coefficient was B = 1.09 per 10 points (*p* = 0.094). The spline test showed no significant non-linearity (*p* = 0.378).

### 3.8. Moderation Analysis

Moderation results are presented in Table 8, with covariate-adjusted subgroup estimates shown in Figure 2 (adjusted for PPOS, DMS, recent legal training, prior malpractice complaint, specialty, experience, and sex, excluding the stratifying variable). The knowledge × recent-training interaction was not significant (B = −0.096, *p* = 0.480), and the knowledge × specialty interaction was also not significant (B = 0.063, *p* = 0.627). Given the sample size, these tests had low power, and the confidence intervals of both interaction terms include values of potential practical relevance. The estimated PRRS difference per 10 knowledge points was 2.95 among physicians without recent legal training and 1.98 among those with recent training.

### 3.9. Exploratory Mediation Analysis

The exploratory parallel mediation analysis is presented in Table 9; the terms below describe the statistical decomposition of the cross-sectional association and do not imply temporal or causal ordering. The total association between legal knowledge and PRRS was 2.94 points per 10 knowledge points (*p* < 0.001), with a direct effect of 1.71 points (*p* = 0.007) and a total indirect effect of 1.23 points (bootstrap 95% CI 0.53–2.19). The indirect effect through PPOS was 0.93 (95% CI 0.22–1.88), whereas the indirect effect through DMS was 0.30 (95% CI −0.05 to 0.92). The indirect association through DMS was therefore compatible with a null or a small positive contribution and does not exclude a modest pathway.

### 3.10. Exploratory Predictive Modeling and Decision-Curve Analysis

The exploratory predictive analyses are shown in Figure 3 (discrimination) and Figure 4 (decision-curve analysis); with approximately four events per predictor, these results represent internal validation only. The apparent AUC was 0.731 (95% CI 0.620–0.842) for legal knowledge alone, 0.804 (95% CI 0.705–0.903) after adding PPOS and DMS, and 0.843 (95% CI 0.752–0.934) for the full eight-predictor model. In DeLong comparisons, the full model had a higher apparent AUC than knowledge alone in this sample (difference 0.112, *p* = 0.018), whereas the intermediate model did not differ significantly from knowledge alone (difference 0.073, *p* = 0.089) or from the full model (difference 0.039, *p* = 0.246). At the Youden-optimal cut-off, the full model had 87.9% sensitivity and 74.6% specificity; the optimism-corrected AUC was 0.789 and the optimism-corrected calibration slope was 0.738. The optimism-corrected calibration slope below 1 suggests appreciable overfitting. Although 1 − 0.738 corresponds to approximately 26% shrinkage, this internal estimate does not establish calibration in new data. In the sensitivity analysis, the direction of results was unchanged with alternative thresholds: with PRRS < 65 (16 events), the optimism-corrected AUC of the full model was 0.752, and with PRRS < 75 (57 events) it was 0.771. In the decision-curve analysis, the full model showed net benefit over the treat-all and treat-none strategies across threshold probabilities of approximately 20–60%, but this apparent benefit was not corrected for optimism and should not be taken as evidence of clinical utility.

## 4. Discussion

### 4.1. Analysis of Findings

This study identified a consistent association between physicians’ legal knowledge and self-reported respect for patients’ rights. The largest group differences involved dignity and privacy, informed consent, and the right to information, while confidentiality scores were comparatively high in both knowledge groups. These findings extend prior evidence showing that formal awareness of patients’ rights does not always translate into reliable bedside implementation [26,27]. They are also consistent with observational work documenting incomplete informed decision-making, confidentiality breaches, and threats to dignity across routine clinical settings [28,29,30]. The integrated PRRS may capture a broader pattern of rights-respecting practice than isolated binary behaviors, which in this sample showed smaller and less precise group differences. An alternative reading, however, deserves equal weight: the six behavioral indicators are closer to verifiable practice, whereas a multi-item composite of frequency ratings is more sensitive to self-presentation, so the divergence between them could reflect social-desirability and common-method effects rather than a broader behavioral pattern [31]. The two readings cannot be separated with self-report data alone, and the PRRS should be interpreted as reported, not observed, practice until it has been compared against chart audit or observation.

A related concern is that the association between legal knowledge and PRRS could partly reflect overlapping content, because both instruments address consent, confidentiality, and information disclosure. Two observations argue against overlap as the sole explanation, without excluding a contribution. First, the knowledge test measures recall of statutory rules (e.g., the age of consent for minors), whereas the PRRS measures the reported frequency of behaviors, so a physician can answer the rules correctly and still not enact them; the moderate correlation (r = 0.479) and the many inadequate-knowledge physicians with high PRRS are consistent with this distinction. Second, the largest group difference was in dignity and privacy (7.3 points, d = 0.82), the one PRRS domain with no counterpart in the knowledge test, whereas confidentiality, which is covered by both instruments, showed the smallest difference (4.0 points). Nevertheless, some shared content and method variance is likely, and the association should be understood as one between legal literacy and reported rights-respecting practice rather than as evidence of a broad effect of legal literacy on actual behavior.

Patient-centered orientation was the strongest independent correlate of PRRS and accounted for the largest component of the indirect association between legal knowledge and rights-respecting practice. This finding suggests one possible interpretation of the legal-literacy association: knowledge of consent, confidentiality, disclosure, and refusal rules may matter most when it is translated into communication behaviors that emphasize shared information, participation, and respect for patient preferences. This interpretation is consistent with the conceptual model outlined in the Introduction, but it is only one of several orderings compatible with cross-sectional data: physicians with a more patient-centered orientation may be more motivated to learn the law that protects patients, or a third factor such as professional conscientiousness may raise both. The indirect association should therefore be regarded as hypothesis-generating rather than as evidence of a causal mechanism. Prior work has linked patient-centered physician behavior with greater patient trust [32], and longitudinal evidence indicates that patient-centered attitudes are modifiable professional characteristics rather than fixed traits [33]. Legal education that is taught through communication scenarios, consent encounters, and shared decision-making exercises may therefore have greater practical value than instruction based primarily on memorization of statutory provisions, a direction supported by recent reviews showing that scenario-based and interactive teaching improves medical students’ legal knowledge and attitudes [16], that interactive approaches improve patient comprehension during consent [34], and that lack of formal training is a principal barrier to shared decision-making in hospitals [35].

Defensive medicine showed a different pattern. Physicians with adequate legal knowledge reported lower defensive behavior, particularly assurance behaviors, yet the indirect association through DMS did not reach statistical significance, a finding that, given the sample size, does not exclude a smaller contribution. This distinction is important because defensive medicine reflects more than a lack of legal knowledge; it is also shaped by malpractice exposure, perceived litigation risk, institutional culture, and the quality of the doctor–patient relationship [14,21,23,24]. Educational interventions should therefore avoid implying that simply reducing defensive behavior will improve respect for patients’ rights. A more balanced objective is to clarify what the law actually requires while reinforcing proportionate documentation, transparent communication, and clinically justified decision-making.

The multivariable and spline analyses further suggest that legal knowledge operates within a broader professional context. Patient-centered orientation, recent legal training, and clinical experience remained independently associated with PRRS, whereas the coefficient for test-measured legal knowledge was attenuated after simultaneous adjustment. The spline analysis did not detect non-linearity (*p* = 0.378), but this does not establish a linear relationship or exclude a threshold effect. At the same time, we found no statistically significant evidence that recent legal training or specialty modified the knowledge–PRRS association; because the interaction tests were underpowered, this absence of evidence should not be read as evidence that no moderation exists. The numerically smaller knowledge slope among recently trained physicians should therefore be viewed as exploratory rather than as evidence of substitution. This pattern is compatible with the literature showing that knowledge is more likely to change clinical behavior when educational content is reinforced by attitudinal and organizational supports [15,36,37].

The subgroup findings were directionally consistent across sex, specialty, experience, recent legal training, and malpractice history, with no statistically significant interaction. Although this consistency supports the general pattern observed in the overall cohort, the subgroup estimates were imprecise, several strata were small (fewer than ten adequate-knowledge physicians among those with <10 years of experience or with prior malpractice exposure), and the number of comparisons performed makes chance findings likely. Larger multicenter samples will be needed to determine whether the association differs meaningfully across specialties or career stages and whether repeated medico-legal training has a measurable effect on rights-respecting behavior over time.

The predictive analyses were exploratory and should be given limited weight. With 33 events and eight predictors, the full model had approximately four events per variable, far below current recommendations [19,20], and the optimism-corrected calibration slope of 0.738 suggests appreciable overfitting; the model also depends on an operational outcome threshold, although the sensitivity analysis suggested that the direction of results did not hinge on the specific cut-off. Bootstrap correction does not substitute for validation in an independent cohort, and neither the discrimination nor the decision-curve results should be taken to imply clinical applicability. At most, the analyses indicate that legal knowledge, patient-centered orientation, and defensive practice carry complementary information about reported practice, a hypothesis that would need to be tested with an adequately sized, externally validated model before any application, such as identifying services that might benefit from focused education, could be considered. Such an approach also recognizes that professional behavior is influenced by system-level factors, including burnout and distress [38], patient empowerment and health literacy [39], and organizational accreditation processes that can embed rights-respecting practices into routine care [40].

### 4.2. Study Limitations

This study has several limitations. Its cross-sectional design precludes causal or temporal ordering, particularly for the mediation analysis, and all principal practice measures were self-reported and therefore susceptible to recall and social-desirability bias. This limitation is particularly important because the primary outcome concerns ethically desirable professional behavior, for which over-reporting is expected to be systematic rather than random; it may inflate absolute PRRS values and, if physicians with more legal knowledge are also more aware of what is expected of them, could inflate the knowledge–PRRS association. In addition, the outcome, the mediator, and the DMS were obtained from the same questionnaire at the same time, so their intercorrelations (e.g., PRRS with PPOS, r = 0.499) are subject to common-method variance [31]; the anonymous format and the separation of the instruments within the questionnaire mitigate but do not remove this. Objective measures such as chart audit, audit of informed-consent documentation, direct observation, standardized-patient encounters, or patient-reported experience were not incorporated because the ethics approval and the participation agreement required anonymity, which precluded linking any external record or observation to an individual respondent, and because observation or standardized-patient assessment across three departments exceeded the resources of this single-center study. The PRRS should therefore be regarded as a measure of reported, not observed, practice. The PRRS was also developed for this study; although preliminary evidence on content validity, internal consistency, and factor structure was reported (Section 2.3), it has not been validated against external criteria or in other samples. The single-center setting also limits generalizability to institutions with different organizational cultures, legal-support structures, or patient populations. The 70-point thresholds for legal knowledge and for suboptimal PRRS, although specified a priori and useful for planned comparisons, remain operational cut-offs rather than externally established boundaries. The large number of secondary, subgroup, interaction, and exploratory tests was not adjusted for multiplicity, so some nominally significant secondary findings may be false positives; only the primary comparison should be regarded as confirmatory, and the mediation, moderation, spline, and predictive analyses are hypothesis-generating.

The sample size was modest, with 96 participants and 33 suboptimal-PRRS events, limiting power for interaction tests and, at approximately four events per variable, placing the logistic prediction model well below accepted sample-size guidance [19,20]. Internal bootstrap correction reduced the apparent discrimination and calibration of the full model, and these results should be regarded as internal validation of an exploratory model with no demonstrated clinical applicability. Finally, only physicians from one academic hospital participated, and the findings may not transfer to non-academic settings or to other legal jurisdictions. Future multicenter and longitudinal studies should combine knowledge testing with objective measures such as chart audit, standardized-patient assessment, direct observation of consent encounters, and repeated evaluation after structured medico-legal and patient-centered communication training.

## 5. Conclusions

Within this single-center cohort, physicians’ legal knowledge was associated with more rights-respecting self-reported practice in unadjusted comparisons, and in exploratory cross-sectional models this association was partly explained statistically by a more patient-centered professional orientation, while defensive medicine accompanied lower knowledge and lower reported respect without statistically conclusive evidence for an indirect association through DMS. The adjusted legal-knowledge coefficient in the full regression model was not statistically significant (*p* = 0.094). Legal literacy, patient-centered orientation, and defensive behavior appear to be distinct but complementary factors relevant to patients’ rights in hospital practice. These associational findings are hypothesis-generating: they suggest that legal education integrated with patient-centered care strategies merits evaluation, but their causal interpretation and any implications for practice await confirmation in larger multicenter, longitudinal studies using objective measures of practice.

## Figures and Tables

**Figure 1:**
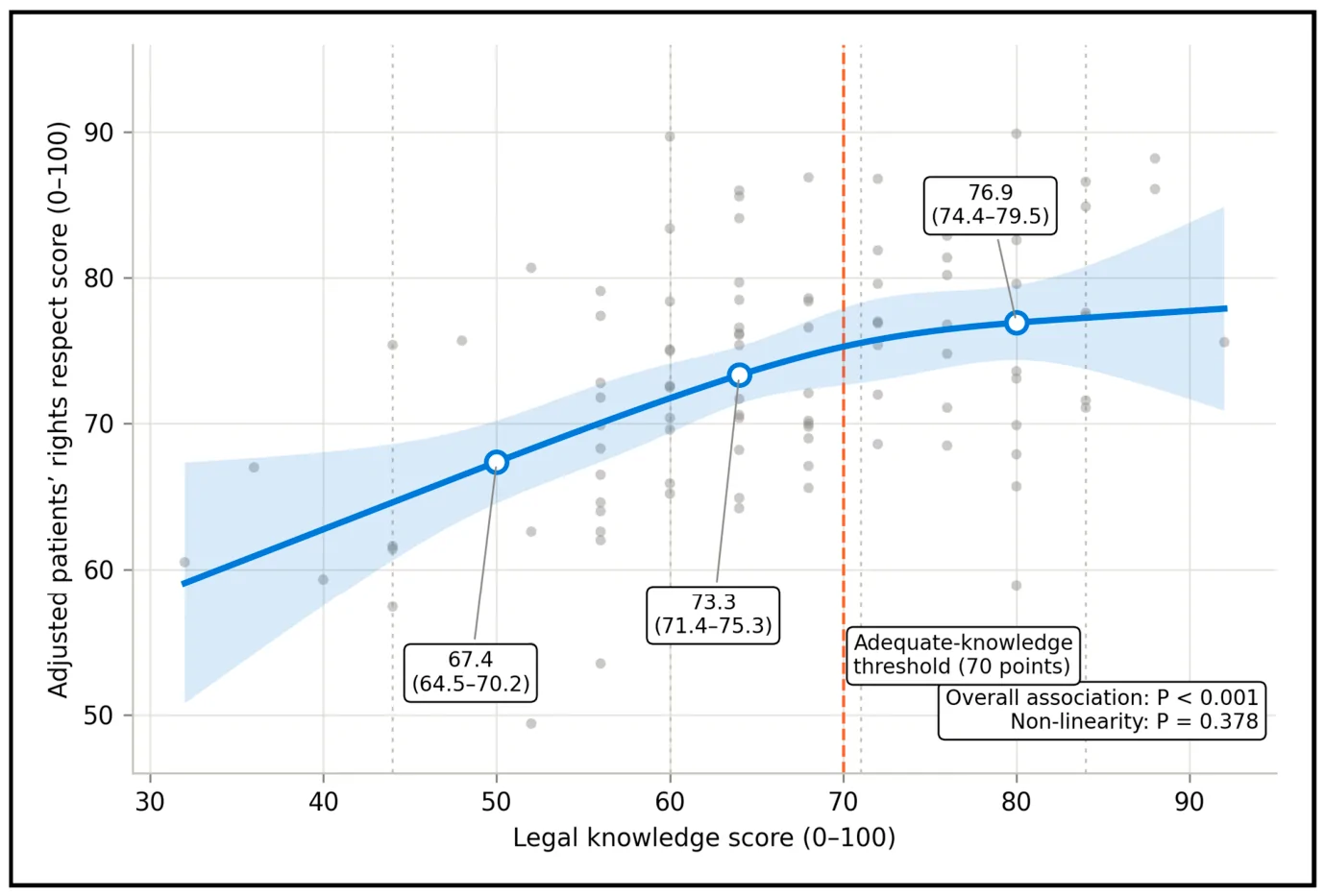
Adjusted Association between Legal Knowledge and PRRS. Abbreviations: PRRS, Patients’ Rights Respect Score; CI, confidence interval. Restricted cubic spline (four knots) with 95% CI, adjusted for PPOS, DMS, recent legal training, prior malpractice complaint, specialty, experience, and sex, with covariates held at their sample means; test for non-linearity *p* = 0.378. Grey dots represent individual physicians; the solid blue line is the adjusted spline estimate and the shaded area its 95% CI; the labelled open circles mark adjusted estimates at selected knowledge scores; the vertical red dashed line indicates the adequate-knowledge threshold (70 points), and the vertical grey dotted lines indicate the positions of the spline knots.

**Figure 2:**
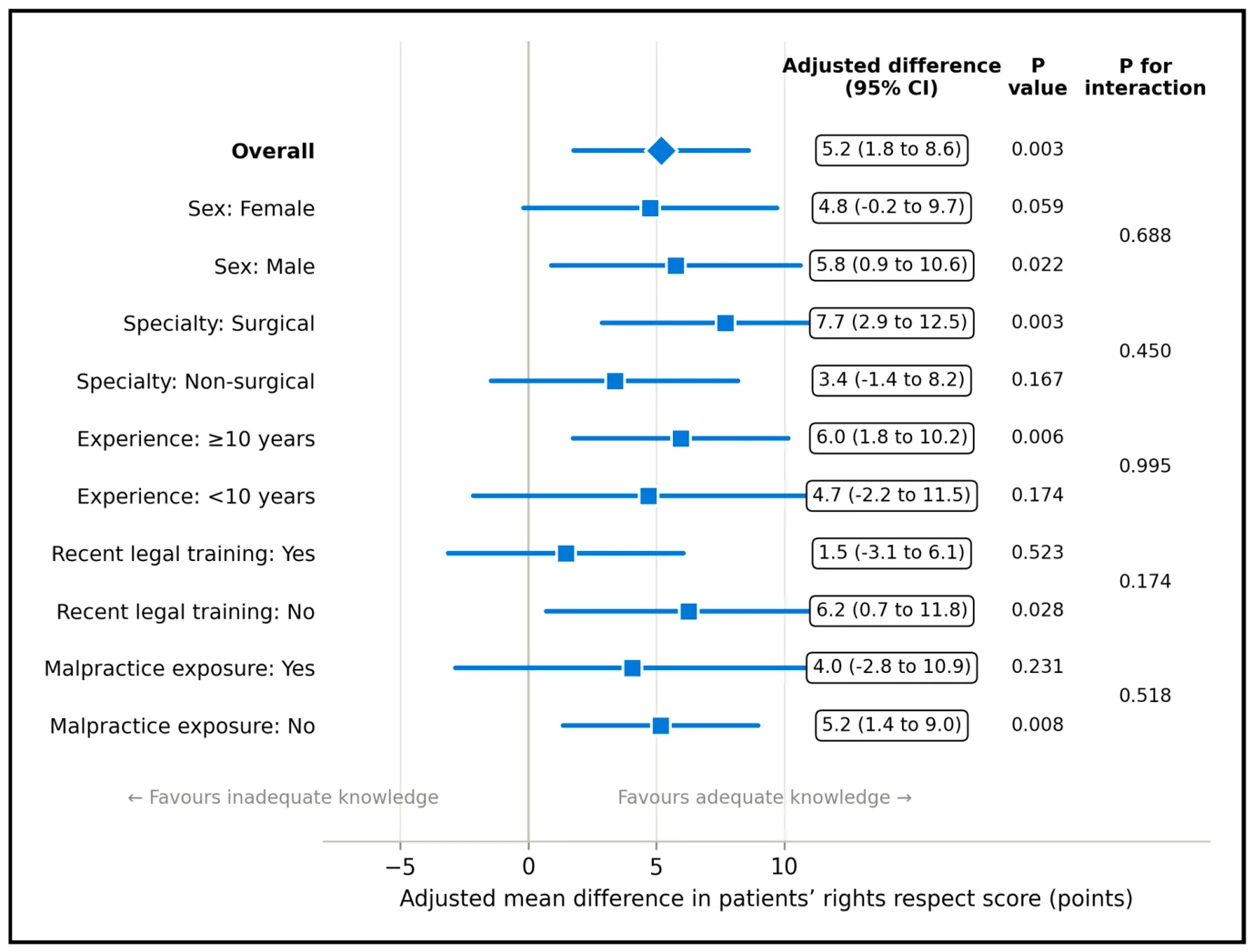
Covariate-Adjusted PRRS Differences by Subgroup. Abbreviations: PRRS, Patients’ Rights Respect Score; CI, confidence interval. Estimates are adjusted for PPOS, DMS, recent legal training, prior malpractice complaint, specialty, experience, and sex (excluding the stratifying variable); *p* for interaction is from the knowledge × subgroup term of the same model. Unadjusted within-stratum estimates are given in Table 5. Squares (subgroups) and the diamond (overall) represent adjusted mean differences, with horizontal lines indicating 95% CIs; the arrows at the bottom of the plot indicate the direction of the difference, favoring inadequate (left) or adequate (right) legal knowledge.

**Figure 3:**
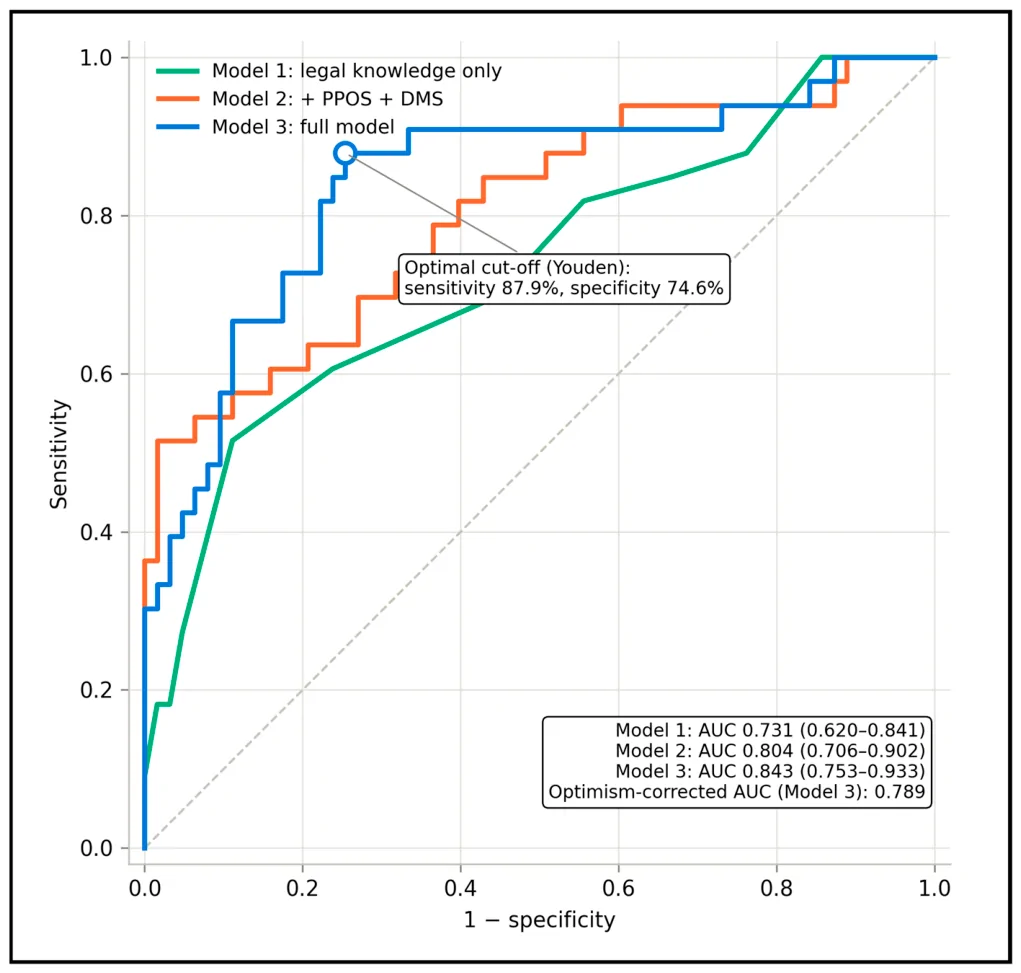
ROC Curves for Suboptimal PRRS (Exploratory Internal Validation). Abbreviations: PRRS, Patients’ Rights Respect Score; PPOS, Patient–Practitioner Orientation Scale; DMS, Defensive Medicine Scale; AUC, area under the curve. Models: legal knowledge alone; legal knowledge, PPOS, and DMS; and the full model (legal knowledge, PPOS, DMS, recent legal training, prior malpractice complaint, surgical specialty, experience, and sex). Apparent AUCs are shown; the optimism-corrected AUC of the full model was 0.789. The dashed diagonal line represents the line of no discrimination (AUC = 0.50), and the open circle marks the Youden-optimal cut-off of the full model.

**Figure 4:**
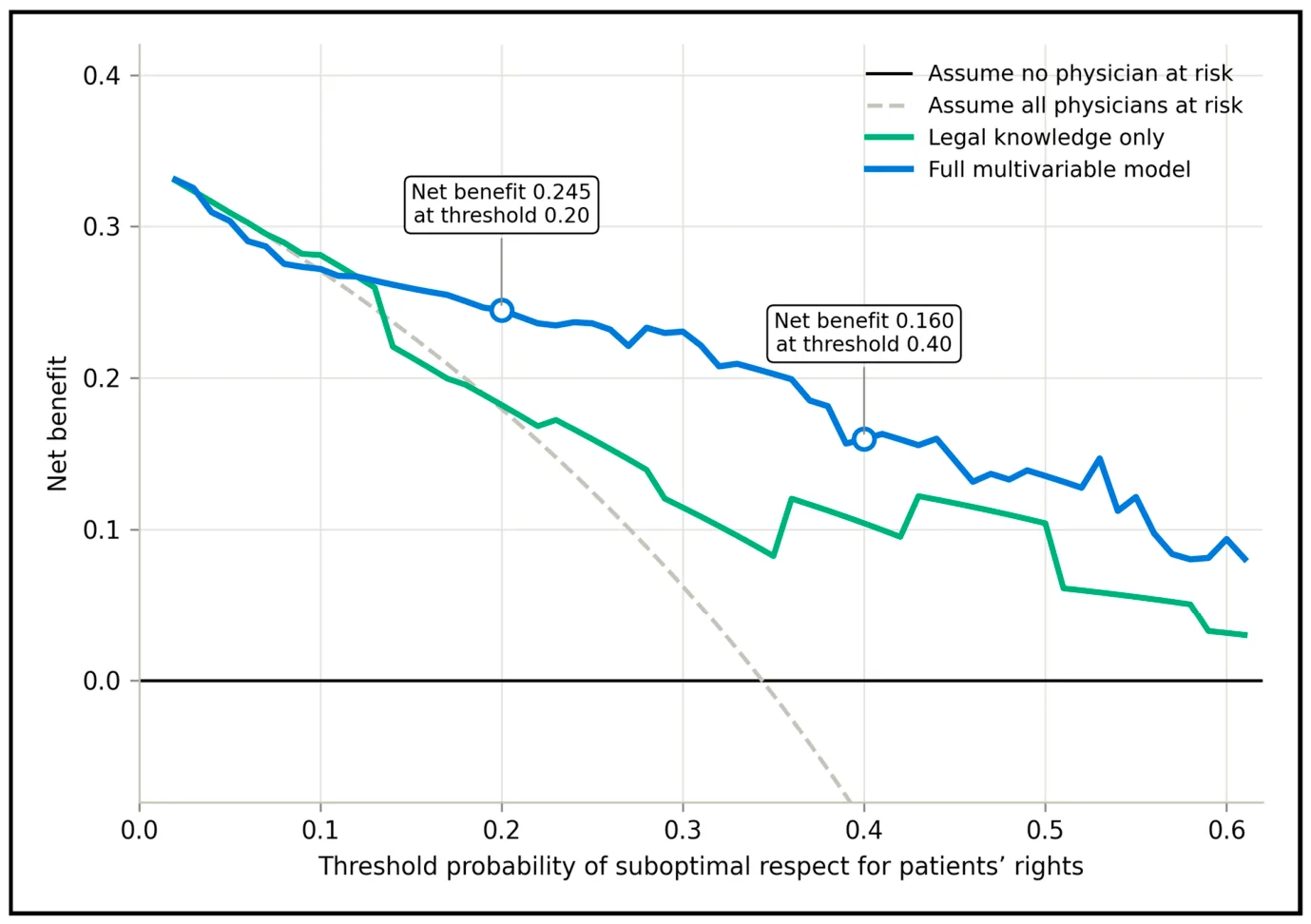
Decision-Curve Analysis (Exploratory). Abbreviations: PRRS, Patients’ Rights Respect Score. Net benefit is shown for the apparent (uncorrected) full model and should be regarded as exploratory; it does not establish clinical utility.

**Table 1:** Baseline Characteristics.

Characteristic	Total (*n* = 96)	Adequate LK (*n* = 34)	Inadequate LK (*n* = 62)	Test Statistic	*p*-Value
Age, years, median (IQR)	40.0 (36–45)	43.0 (38–48)	38.5 (34–44)	U = 1360.0	0.019
Female sex, *n* (%)	54 (56.2)	19 (55.9)	35 (56.5)	χ^2^ = 0.003	0.957
Specialty profile, *n* (%)				χ^2^ = 5.80	0.055
Medical	40 (41.7)	9 (26.5)	31 (50.0)		
Surgical	37 (38.5)	15 (44.1)	22 (35.5)		
Emergency/intensive care	19 (19.8)	10 (29.4)	9 (14.5)		
Professional grade, *n* (%)				χ^2^ = 4.69	0.096
Resident	24 (25.0)	6 (17.6)	18 (29.0)		
Specialist	35 (36.5)	10 (29.4)	25 (40.3)		
Senior physician	37 (38.5)	18 (52.9)	19 (30.6)		
Experience, years, median (IQR)	12.0 (7–16)	14.5 (10–21)	10.0 (5–15)	U = 1396.0	0.009
Recent legal training, *n* (%)	43 (44.8)	23 (67.6)	20 (32.3)	χ^2^ = 11.12	<0.001
Formal law/ethics course during training, *n* (%)	71 (74.0)	29 (85.3)	42 (67.7)	χ^2^ = 3.51	0.061
Medico-legal training hours, past 5 years, median (IQR)	4 (0–12)	8 (0–16)	0 (0–8)	U = 1421.5	0.003
Prior malpractice complaint, *n* (%)	20 (20.8)	6 (17.6)	14 (22.6)	χ^2^ = 0.32	0.569
Legal knowledge score, mean ± SD	65.9 ± 12.1	78.8 ± 5.4	58.8 ± 8.3	—	—

Abbreviations: LK, legal knowledge; IQR, interquartile range; SD, standard deviation; U, Mann–Whitney U statistic; χ^2^, chi-square statistic.

**Table 2:** Patients’ Rights Respect Scores.

Outcome (0–100)	Adequate LK (*n* = 34), Mean ± SD	Inadequate LK (*n* = 62), Mean ± SD	MD (95% CI)	t (df)	*p*-Value	Cohen’s d
PRRS, total	76.8 ± 7.1	71.0 ± 8.4	5.8 (2.6 to 9.0)	3.60 (78.5)	<0.001	0.73
Informed consent	76.3 ± 8.3	70.2 ± 9.7	6.1 (2.4 to 9.9)	3.26 (77.4)	0.002	0.66
Confidentiality	78.7 ± 8.6	74.7 ± 9.9	4.0 (0.2 to 7.9)	2.08 (76.0)	0.041	0.43
Right to information	71.8 ± 8.8	65.9 ± 9.8	5.8 (1.9 to 9.7)	2.98 (74.4)	0.004	0.62
Dignity and privacy	80.4 ± 8.5	73.2 ± 9.1	7.3 (3.6 to 10.9)	3.91 (72.1)	<0.001	0.82

Abbreviations: PRRS, Patients’ Rights Respect Score; LK, legal knowledge; SD, standard deviation; MD, mean difference; CI, confidence interval; df, degrees of freedom.

**Table 3:** Defensive Medicine and Patient-Centered Orientation.

Outcome (Range)	Adequate LK (*n* = 34), Mean ± SD	Inadequate LK (*n* = 62), Mean ± SD	MD (95% CI)	t (df)	*p*-Value	Cohen’s d
DMS, total (15–75)	44.5 ± 6.8	48.5 ± 9.3	−4.1 (−7.4 to −0.8)	−2.45 (86.3)	0.016	−0.48
Assurance behaviors (9–45)	26.9 ± 4.2	29.6 ± 6.6	−2.7 (−4.9 to −0.5)	−2.46 (92.2)	0.016	−0.46
Avoidance behaviors (6–30)	17.6 ± 3.6	18.9 ± 3.6	−1.4 (−2.9 to 0.2)	−1.77 (68.7)	0.082	−0.38
PPOS, total (1–6)	4.34 ± 0.52	4.15 ± 0.49	0.18 (−0.04 to 0.40)	1.67 (64.2)	0.101	0.36
Sharing subscale (1–6)	4.32 ± 0.51	4.13 ± 0.55	0.20 (−0.03 to 0.42)	1.76 (72.0)	0.082	0.37
Caring subscale (1–6)	4.35 ± 0.67	4.18 ± 0.55	0.16 (−0.11 to 0.43)	1.21 (57.3)	0.230	0.28

Abbreviations: DMS, Defensive Medicine Scale; PPOS, Patient–Practitioner Orientation Scale; LK, legal knowledge; SD, standard deviation; MD, mean difference; CI, confidence interval; df, degrees of freedom.

**Table 4:** Rights-Related Practice Indicators.

Practice Indicator	Total, *n* (%)	Adequate LK, *n* (%)	Inadequate LK, *n* (%)	OR (95% CI)	χ^2^	*p*-Value
Always obtains written informed consent before invasive procedures	71 (74.0)	28 (82.4)	43 (69.4)	2.06 (0.73–5.80)	1.93	0.165
Routinely verifies patient understanding (teach-back)	40 (41.7)	17 (50.0)	23 (37.1)	1.70 (0.73–3.96)	1.50	0.220
Systematically presents alternatives, including the option of refusal	56 (58.3)	21 (61.8)	35 (56.5)	1.25 (0.53–2.93)	0.26	0.614
Has disclosed clinical information to family without patient consent	40 (41.7)	11 (32.4)	29 (46.8)	0.54 (0.23–1.31)	1.88	0.170
Systematically documents informed refusal of treatment	65 (67.7)	23 (67.6)	42 (67.7)	1.00 (0.41–2.44)	<0.01	0.992
Aware of the hospital patients’ rights procedure	46 (47.9)	18 (52.9)	28 (45.2)	1.37 (0.59–3.16)	0.53	0.466

Abbreviations: LK, legal knowledge; OR, odds ratio; CI, confidence interval; χ^2^, chi-square statistic. For the indicator “Has disclosed clinical information to family without patient consent”, the direction is reversed relative to the other indicators: a lower proportion and an OR < 1 indicate practice more respectful of patients’ rights.

**Table 5:** Unadjusted Subgroup Analyses.

Subgroup	*n* (Adequate/Inadequate)	Adequate LK, Mean ± SD	Inadequate LK, Mean ± SD	MD (95% CI)	*p*-Value	*p* for Interaction
Sex: female	19/35	77.6 ± 7.8	72.3 ± 8.7	5.2 (0.5 to 9.9)	0.030	0.688
Sex: male	15/27	75.9 ± 6.1	69.3 ± 7.9	6.6 (2.2 to 11.0)	0.005	
Specialty: surgical	15/22	77.7 ± 6.1	70.4 ± 8.0	7.4 (2.7 to 12.1)	0.003	0.450
Specialty: non-surgical	19/40	76.1 ± 7.8	71.3 ± 8.7	4.7 (0.2 to 9.3)	0.043	
Experience: ≥10 years	26/32	76.9 ± 7.3	71.2 ± 8.4	5.7 (1.6 to 9.8)	0.008	0.995
Experience: <10 years †	8/30	76.4 ± 6.7	70.8 ± 8.5	5.7 (−0.4 to 11.8)	0.066	
Recent legal training: yes	23/20	77.4 ± 6.0	75.4 ± 8.6	2.0 (−2.7 to 6.7)	0.397	0.174
Recent legal training: no	11/42	75.6 ± 9.1	68.9 ± 7.5	6.7 (0.3 to 13.2)	0.041	
Malpractice exposure: yes †	6/14	70.8 ± 6.6	67.4 ± 5.7	3.4 (−3.7 to 10.4)	0.306	0.518
Malpractice exposure: no	28/48	78.1 ± 6.6	72.0 ± 8.8	6.1 (2.5 to 9.6)	0.001	

Abbreviations: LK, legal knowledge; SD, standard deviation; MD, mean difference; CI, confidence interval. Estimates are unadjusted within-stratum comparisons (Welch *t*-test); *p* for interaction derives from the knowledge × subgroup term in the covariate-adjusted model (adjusted for PPOS, DMS, recent legal training, prior malpractice complaint, specialty, experience, and sex, excluding the stratifying variable) and is therefore identical to that reported in Section 3.8. † Stratum with fewer than 10 adequate-knowledge physicians; the estimate is imprecise and should not be interpreted.

**Table 6:** Correlations among Continuous Measures.

Variable	1	2	3	4	5	6
1. Legal knowledge score	—					
2. PRRS	0.479 (<0.001)	—				
3. PPOS	0.319 (0.002)	0.499 (<0.001)	—			
4. DMS	−0.320 (0.001)	−0.427 (<0.001)	−0.186 (0.069)	—		
5. Experience, years	0.275 (0.007)	0.190 (0.063)	−0.059 (0.567)	−0.040 (0.699)	—	
6. Age, years	0.277 (0.006)	0.224 (0.028)	−0.066 (0.523)	−0.058 (0.572)	0.968 (<0.001)	—

Abbreviations: PRRS, Patients’ Rights Respect Score; PPOS, Patient–Practitioner Orientation Scale; DMS, Defensive Medicine Scale. Values are Pearson r (*p*-value); column numbers 1–6 correspond to the numbered row variables, and only the lower triangle is shown.

**Table 7:** Multivariable Regression.

Predictor	B	95% CI	β	t	*p*-Value
Legal knowledge, per 10 points	1.09	−0.19 to 2.36	0.157	1.69	0.094
PPOS, per 1 point	6.64	3.95 to 9.32	0.399	4.91	<0.001
DMS, per 10 points	−1.67	−3.67 to 0.32	−0.174	−1.67	0.099
Recent legal training	3.89	1.18 to 6.60	0.232	2.86	0.005
Prior malpractice complaint	−2.28	−6.33 to 1.77	−0.111	−1.12	0.266
Surgical specialty	0.22	−2.38 to 2.83	0.013	0.17	0.866
Experience, per 10 years	1.76	0.16 to 3.36	0.178	2.18	0.032
Female sex	1.54	−1.06 to 4.15	0.092	1.18	0.242

Model fit: R^2^ = 0.513, adjusted R^2^ = 0.468, F(8, 87) = 11.46, *p* < 0.001, RMSE = 6.1; Breusch–Pagan χ^2^(8) = 9.84, *p* = 0.276; maximum VIF = 1.94. Abbreviations: B, unstandardized coefficient; CI, confidence interval; β, standardized coefficient; PPOS, Patient–Practitioner Orientation Scale; DMS, Defensive Medicine Scale; RMSE, root mean square error; VIF, variance inflation factor.

**Table 8:** Moderation Analysis.

Model/Term	B (95% CI)	*p*-Value	Model R^2^	ΔR^2^	*p* for ΔF
Model 1 (main effects)			0.307	—	—
Legal knowledge, per point	0.260 (0.126 to 0.394)	<0.001			
Recent legal training	3.74 (0.60 to 6.88)	0.020			
Model 2 (+knowledge × training)			0.311	0.004	0.480
Knowledge × training	−0.096 (−0.366 to 0.173)	0.480			
Model 3 (+knowledge × specialty)			0.313	0.002	0.627
Knowledge × surgical specialty	0.063 (−0.194 to 0.320)	0.627			
Simple slope: no recent training	2.95 (1.29 to 4.60)	<0.001			
Simple slope: recent training	1.98 (−0.20 to 4.16)	0.075			

Abbreviations: B, unstandardized coefficient; CI, confidence interval; ΔR^2^, change in R^2^; ΔF, F-test of the R^2^ change.

**Table 9:** Exploratory Mediation Analysis.

Path/Effect	B	95% CI	*p*-Value
a_1_: knowledge → PPOS	0.148	0.064 to 0.233	<0.001
a_2_: knowledge → DMS (per 10 points)	−0.185	−0.299 to −0.072	0.002
b_1_: PPOS → PRRS	6.28	3.51 to 9.04	<0.001
b_2_: DMS → PRRS	−1.60	−3.65 to 0.45	0.124
Total effect (c)	2.94	1.70 to 4.18	<0.001
Direct effect (c′)	1.71	0.48 to 2.95	0.007
Indirect effect via PPOS (a_1_b_1_)	0.93	0.22 to 1.88	—
Indirect effect via DMS (a_2_b_2_)	0.30	−0.05 to 0.92	—
Total indirect effect	1.23	0.53 to 2.19	—
Proportion mediated	41.8%	—	—

Abbreviations: PPOS, Patient–Practitioner Orientation Scale; DMS, Defensive Medicine Scale; PRRS, Patients’ Rights Respect Score; B, unstandardized coefficient; CI, confidence interval. Direct, indirect, and total “effects” are statistical decomposition terms for a cross-sectional association; the reverse ordering (patient-centered orientation promoting the acquisition of legal knowledge) is equally compatible with these data.

## Data Availability

The data presented in this study are available from the corresponding author upon reasonable request; participant-level data are not publicly available due to privacy and ethical restrictions.

## Abbreviations

The following abbreviations are used in this manuscript: PRRS, Patients’ Rights Respect Score; LK, legal knowledge; DMS, Defensive Medicine Scale; PPOS, Patient–Practitioner Orientation Scale; SD, standard deviation; IQR, interquartile range; MD, mean difference; CI, confidence interval; OR, odds ratio; AUC, area under the curve; RMSE, root mean square error; VIF, variance inflation factor; ROC, receiver operating characteristic; CVI, content validity index; STROBE, Strengthening the Reporting of Observational Studies in Epidemiology.

## References

[1] Fallberg L., Mackenney S. (2003). Patient Ombudsmen in seven European countries: An effective way to implement patients’ rights?. Eur. J. Health Law.

[2] Ducinskiene D., Vladickiene J., Kalediene R., Haapala I. (2006). Awareness and practice of patient’s rights law in Lithuania. BMC Int. Health Hum. Rights.

[3] Kuzu N., Ergin A., Zencir M. (2006). Patients’ awareness of their rights in a developing country. Public Health.

[4] Merakou K., Dalla-Vorgia P., Garanis-Papadatos T., Kourea-Kremastinou J. (2001). Satisfying patients’ rights: A hospital patient survey. Nurs. Ethics.

[5] White B., Willmott L., Cartwright C., Parker M.H., Williams G. (2014). Doctors’ knowledge of the law on withholding and withdrawing life-sustaining medical treatment. Med. J. Aust..

[6] Hariharan S., Jonnalagadda R., Walrond E., Moseley H. (2006). Knowledge, attitudes and practice of healthcare ethics and law among doctors and nurses in Barbados. BMC Med. Ethics.

[7] Preston-Shoot M., McKimm J. (2011). Towards effective outcomes in teaching, learning and assessment of law in medical education. Med. Educ..

[8] Studdert D.M., Mello M.M., Sage W.M., DesRoches C.M., Peugh J., Zapert K., Brennan T.A. (2005). Defensive medicine among high-risk specialist physicians in a volatile malpractice environment. JAMA.

[9] Ortashi O., Virdee J., Hassan R., Mutrynowski T., Abu-Zidan F. (2013). The practice of defensive medicine among hospital doctors in the United Kingdom. BMC Med. Ethics.

[10] Vento S., Cainelli F., Vallone A. (2018). Defensive medicine: It is time to finally slow down an epidemic. World J. Clin. Cases.

[11] Stewart M., Brown J.B., Donner A., McWhinney I.R., Oates J., Weston W.W., Jordan J. (2000). The impact of patient-centered care on outcomes. J. Fam. Pract..

[12] Krupat E., Rosenkranz S.L., Yeager C.M., Barnard K., Putnam S.M., Inui T.S. (2000). The practice orientations of physicians and patients: The effect of doctor–patient congruence on satisfaction. Patient Educ. Couns..

[13] Ajzen I. (1991). The theory of planned behavior. Organ. Behav. Hum. Decis. Process..

[14] Baungaard N., Skovvang P.L., Assing Hvidt E., Gerbild H., Kirstine Andersen M., Lykkegaard J. (2022). How defensive medicine is defined in European medical literature: A systematic review. BMJ Open.

[15] Plaiasu M.C., Alexandru D.O., Nanu C.A. (2022). Physicians’ legal knowledge of informed consent and confidentiality. A cross-sectional study. BMC Med. Ethics.

[16] Abbasi M., Shirazi M., Torkmandi H., Homayoon S., Abdi M. (2023). Impact of teaching, learning, and assessment of medical law on cognitive, affective and psychomotor skills of medical students: A systematic review. BMC Med. Educ..

[17] Plaiasu M.C., Alexandru D.O., Nanu C.A. (2023). Patients’ rights in physicians’ practice during Covid-19 pandemic: A cross-sectional study in Romania. BMC Med. Ethics.

[18] von Elm E., Altman D.G., Egger M., Pocock S.J., Gøtzsche P.C., Vandenbroucke J.P. (2007). The Strengthening the Reporting of Observational Studies in Epidemiology (STROBE) statement: Guidelines for reporting observational studies. Lancet.

[19] Riley R.D., Ensor J., Snell K.I.E., Harrell F.E., Martin G.P., Reitsma J.B., Moons K.G.M., Collins G., van Smeden M. (2020). Calculating the sample size required for developing a clinical prediction model. BMJ.

[20] Peduzzi P., Concato J., Kemper E., Holford T.R., Feinstein A.R. (1996). A simulation study of the number of events per variable in logistic regression analysis. J. Clin. Epidemiol..

[21] Asher E., Greenberg-Dotan S., Halevy J., Glick S., Reuveni H. (2012). Defensive medicine in Israel—A nationwide survey. PLoS ONE.

[22] Polit D.F., Beck C.T. (2006). The content validity index: Are you sure you know what’s being reported? Critique and recommendations. Res. Nurs. Health.

[23] Jena A.B., Seabury S., Lakdawalla D., Chandra A. (2011). Malpractice risk according to physician specialty. N. Engl. J. Med..

[24] He A.J. (2014). The doctor–patient relationship, defensive medicine and overprescription in Chinese public hospitals: Evidence from a cross-sectional survey in Shenzhen city. Soc. Sci. Med..

[25] Preacher K.J., Hayes A.F. (2008). Asymptotic and resampling strategies for assessing and comparing indirect effects in multiple mediator models. Behav. Res. Methods.

[26] Sheikhtaheri A., Jabali M.S., Dehaghi Z.H. (2016). Nurses’ knowledge and performance of the patients’ bill of rights. Nurs. Ethics.

[27] Dessalegn K., Girma B., Oumer K.E., Hunie M., Belete K.G. (2022). Patients’ awareness of their rights, associated factors and its practice by health professionals from a patient perspective among elective surgical patients at Tikur Anbessa Specialized Hospital, Addis Ababa, Ethiopia: A cross-sectional study, 2021. BMJ Open.

[28] Braddock C.H., Edwards K.A., Hasenberg N.M., Laidley T.L., Levinson W. (1999). Informed decision making in outpatient practice: Time to get back to basics. JAMA.

[29] Beltran-Aroca C.M., Girela-Lopez E., Collazo-Chao E., Montero-Pérez-Barquero M., Muñoz-Villanueva M.C. (2016). Confidentiality breaches in clinical practice: What happens in hospitals?. BMC Med. Ethics.

[30] Baillie L. (2009). Patient dignity in an acute hospital setting: A case study. Int. J. Nurs. Stud..

[31] Podsakoff P.M., MacKenzie S.B., Podsakoff N.P. (2012). Sources of method bias in social science research and recommendations on how to control it. Annu. Rev. Psychol..

[32] Fiscella K., Meldrum S., Franks P., Shields C.G., Duberstein P., McDaniel S.H., Epstein R.M. (2004). Patient trust: Is it related to patient-centered behavior of primary care physicians?. Med. Care.

[33] Tsimtsiou Z., Kerasidou O., Efstathiou N., Papaharitou S., Hatzimouratidis K., Hatzichristou D. (2007). Medical students’ attitudes toward patient-centred care: A longitudinal survey. Med. Educ..

[34] Glaser J., Nouri S., Fernandez A., Sudore R.L., Schillinger D., Klein-Fedyshin M., Schenker Y. (2020). Interventions to improve patient comprehension in informed consent for medical and surgical procedures: An updated systematic review. Med. Decis. Mak..

[35] Waddell A., Lennox A., Spassova G., Bragge P. (2021). Barriers and facilitators to shared decision-making in hospitals from policy to practice: A systematic review. Implement. Sci..

[36] Cabana M.D., Rand C.S., Powe N.R., Wu A.W., Wilson M.H., Abboud P.A., Rubin H.R. (1999). Why don’t physicians follow clinical practice guidelines? A framework for improvement. JAMA.

[37] Cunningham N., Pham T., Kennedy B., Gillard A., Ibrahim J. (2017). A cross-sectional survey using electronic distribution of a questionnaire to subscribers of educational material written by clinicians, for clinicians, to evaluate whether practice change resulted from reading the Clinical Communiqué. BMJ Open.

[38] Shanafelt T.D., Balch C.M., Bechamps G., Russell T., Dyrbye L., Satele D., Collicott P., Novotny P.J., Sloan J., Freischlag J. (2010). Burnout and medical errors among American surgeons. Ann. Surg..

[39] Schulz P.J., Nakamoto K. (2013). Health literacy and patient empowerment in health communication: The importance of separating conjoined twins. Patient Educ. Couns..

[40] Greenfield D., Braithwaite J. (2008). Health sector accreditation research: A systematic review. Int. J. Qual. Health Care.

